# Decoding of top-down cognitive processing for SSVEP-controlled BMI

**DOI:** 10.1038/srep36267

**Published:** 2016-11-03

**Authors:** Byoung-Kyong Min, Sven Dähne, Min-Hee Ahn, Yung-Kyun Noh, Klaus-Robert Müller

**Affiliations:** 1 Department of Brain and Cognitive Engineering, Korea University, Seoul 02841, Republic of Korea; 2 Machine Learning Group, Berlin Institute of Technology, Berlin 10587, Germany; 3 Department of Mechanical and Aerospace Engineering, Seoul National University, Seoul 08826, Republic of Korea

## Abstract

We present a fast and accurate non-invasive brain-machine interface (BMI) based on demodulating steady-state visual evoked potentials (SSVEPs) in electroencephalography (EEG). Our study reports an SSVEP-BMI that, for the first time, decodes primarily based on *top-down* and not *bottom-up* visual information processing. The experimental setup presents a grid-shaped flickering line array that the participants observe while intentionally attending to a subset of flickering lines representing the shape of a letter. While the flickering pixels stimulate the participant’s visual cortex uniformly with equal probability, the participant’s intention groups the strokes and thus perceives a ‘letter Gestalt’. We observed decoding accuracy of 35.81% (up to 65.83%) with a regularized linear discriminant analysis; on average 2.05-fold, and up to 3.77-fold greater than chance levels in multi-class classification. Compared to the EEG signals, an electrooculogram (EOG) did not significantly contribute to decoding accuracies. Further analysis reveals that the top-down SSVEP paradigm shows the most focalised activation pattern around occipital visual areas; Granger causality analysis consistently revealed prefrontal top-down control over early visual processing. Taken together, the present paradigm provides the first neurophysiological evidence for the top-down SSVEP BMI paradigm, which potentially enables multi-class intentional control of EEG-BMIs without using gaze-shifting.

BMIs allow a systematic decoding of brain states for communication, control, and monitoring^1^,^2^,^3^,^4^. Among several neuroimaging techniques, EEG has been shown to be a practical and versatile tool for operating general BMIs because of its excellent temporal resolution (approximately 1 ms), non-invasiveness, and portability^3^,^5^,^6^. EEG-based BMI studies^7^ have established a number of frequently used robust paradigms, including event-related P300^8^,^9^, slow cortical potential^10^, mu rhythm^11^,^12^, and SSVEPs^13^. Among these paradigms, SSVEP-BMIs provide very accurate high temporal and spectral resolution information (usually less than 0.1 Hz)^14^ at high information transfer rates (ITRs)^15^; depending on the number of classes, even though ITR is high, accuracy can still be too low for effective communication^16^. An SSVEP is a physically driven electrical oscillatory response in the brain, induced by the repetitive presentation of a visual stimulus^14^; occipital SSVEP can be detected at the same flicker frequency (and harmonics) as the presented flickering stimulus, a paradigm that has many applications in BMI and neurotechnology^17^,^18^,^19^. The basic concept for an SSVEP-mediated BMI system dates back to the late 1970s^20^, and the application of SSVEPs to BMIs was introduced almost 20 years later^21^. The SSVEP-BMI has been further generalized to encode user commands in flickering stimuli that induce SSVEPs at different frequencies. The user chooses one of the commands by focusing attention on one of the oscillating stimuli, and by analyzing the subsequent SSVEPs, the BMI system tries to decode which stimulus the user chose^15^,^22^. As SSVEP signals are triggered by external stimuli, which are more robust and easier to control than internally generated stimuli, SSVEPs can be potent and stable BMI control signals. Moreover, SSVEP-BMI systems can be used by more than 90% of users without much training^22^,^23^. Nevertheless, SSVEP-based BMIs still have several limitations to overcome. For instance, the flicker stimulation can produce visual fatigue or discomfort^24^. In addition, most SSVEP-based BMIs are not free from gaze-shift issues^25^,^26^,^27^,^28^. Therefore, an SSVEP-based BMI is usually regarded as a dependent BMI, because it requires some neuromuscular control of the eyes and/or head^29^. In addition, there is a limit to the number of classes that are reliably decodable using conventional EEG-based BMIs^30^,^31^. Therefore, a breakthrough allowing the decoding of multi-class intention with sufficient accuracy would be highly welcomed.

In the present study, we advance the classical (occipital or bottom-up) SSVEP paradigm^15^,^22^,^32^,^33^,^34^,^35^,^36^, allowing a greater variety of human intentional top-down processes to be identified in a significantly more subtle manner for intentional BMI control. For this purpose, we introduce a flickering grid-shaped line array (Fig. 1 and Supplementary video clip 1), from which the shape of the symbol intended to be communicated (e.g., different letters, numbers, or symbols) can be perceived by the study participant as a “symbol Gestalt” using selective visuospatial attention. *Gestalt* means “the shape of the whole”, and the underlying assumption of the Gestalt school of thought is that psychological phenomena are better understood when viewed as organized wholes, rather than when broken down into their component parts^37^. Before the rise of post-behaviorist cognitive psychology, the Gestalt psychologists (who actively emerged from Germany in the 1920s to 1940s) resisted behaviorism, with this differing approach to understanding behavior. A user places his/her attention on the randomly flickering sets of pixels and his/her brain *constructs* the desired percept by top-down processing, i.e., the specific shape is *cognitively* formed from combinations of flickering line configurations (for an overview, see Fig. 1b). Although most SSVEP-based BMI studies have so far employed a single stimulation frequency to encode each selection, in the present study, we adopt a multiple-frequency stimulation method. In earlier studies, dual-frequency^38^, three- or four-frequency^19^,^39^, and more-than-four-frequency^27^,^28^,^36^ stimulations have been proposed to enhance SSVEP-based BMI information transfer, but these methods are still restricted in their ability to express a variety of symbols and are accompanied by unavoidable gaze-shift. In principle, symbols or letters can be encoded by a combination of selected line segments (individually flickering at their own frequencies), which are components of the SSVEP-inducing grid-shaped line array. In order to demonstrate the proposed principle for an example with six stimulus categories, we suggest a machine learning decoding framework that can resolve multiple top-down modulated responses to external stimulation. Using a cross-validation method^40^, new, previously unseen EEG trials in the prediction phase were classified based on extracted (hyper-) parameters from labelled training EEG trials in the calibration phase (see Fig. 2 for the workflow). We then compared the decoding accuracies, activation patterns, and directed functional connectivities of the newly proposed top-down SSVEP condition with those of a classical SSVEP condition (that we refer to as bottom-up; see Supplementary video clip 3). To investigate an intermediate effect between these top-down and bottom-up conditions, we included an intermediate experimental condition, in which the luminance of task-irrelevant flickering lines is reduced by 1/10 of the original (see Supplementary video clip 2). Although changes in the luminance of task-irrelevant flickering lines still require knowledge of the target stimulus (i.e., task-relevant flickering lines), we anticipated gradual (or systematic) changes in both decoding accuracies and neurophysiological EEG measures from bottom-up to intermediate and from intermediate to top-down conditions. Therefore, although the intermediate condition is irrelevant for BMI-mediated free communication, we introduce it in the present study.

## Results

### Decoding accuracy

Twenty healthy volunteers participated in this study, which consisted of the three experimental conditions (i.e., top-down, intermediate, and bottom-up SSVEP conditions). Each experimental condition had six classes of stimuli (see Table 1), and each class was composed of 40 trials. Consequently, each experimental condition had 240 trials. Although the chance level is theoretically 21.19% based on these parameters^41^, we designate the empirical chance level for the present study as the six-class choice accuracy computed by randomly shuffling all the obtained data, which is 17.44% on average. We observed significant differences in decoding accuracies across the classification models for all three experimental conditions: top-down SSVEP (*F*(3,57) = 49.684, *p* < 0.0001), intermediate SSVEP (*F*(3,57) = 81.938, *p* < 0.0001), and bottom-up SSVEP (*F*(3,57) = 138.197, *p* < 0.0001). Post hoc comparisons revealed that our novel top-down SSVEP paradigm for multi-class decoding of EEG signals by regularized linear discriminant analysis (rLDA) with shrinkage^8^,^40^,^42^ performed well; i.e., classification was, on average, 2.05-fold (35.81%) and as high as 3.77-fold (65.83%) greater than chance (i.e., 17.44%; *t*(19) = 7.283, *p* < 0.0001, false discovery rate [FDR] corrected). The decoding accuracies from each classification model (i.e., rLDA of EEG signals, randomly-shuffled rLDA of EEG signals, rLDA of EOG signals, and canonical correlation analysis (CCA)^43^ of EEG signals) were statistically compared using two-tailed paired *t*-tests, and multiple comparisons were corrected using the FDR (detailed in the *Analysis method* section). When the EEG signals from just three occipital electrodes were computed, the decoding accuracy was significantly enhanced (42.52%; *t*(19) = −2.685, *p* < 0.05). In comparison, CCA of the EEG signals yielded decoding accuracies that were not significantly different from chance (19.23%; *t*(19) = −1.403, *n.s.*, FDR corrected). Therefore, it is noteworthy that LDA provided significantly improved decoding accuracies in the top-down condition compared to CCA (*t*(19) = 7.955, *p* < 0.0001, FDR corrected). When using LDA to analyze EOG signals, decoding accuracies were not significantly different from chance (*t*(19) = 0.742, *n.s.*, FDR corrected). Therefore, EOG signals did not significantly contribute to decoding accuracies in the top-down SSVEP condition. In accordance with this observation, decoding accuracies by LDA of EOG signals (16.69%) were significantly lower than those by LDA of EEG signals (35.81%) in the top-down SSVEP condition (*t*(19) = 8.445, *p* < 0.0001, FDR corrected).

As shown in Fig. 3a, systematic enhancement of classification accuracies by LDA was observed from top-down (35.81%) to intermediate (56.73%) and finally to bottom-up condition (75.44%; *F*(2,57) = 38.885, *p* < 0.001), which were all significantly greater than chance. The decoding accuracies of the EEG signals from only the three occipital electrodes were significantly increased in the intermediate condition (60.02%; *t*(19) = −2.094, *p* < 0.05) but not in the bottom-up condition (73.79%; *t*(19) = 0.855, *n.s.*). The decoding accuracies using EOG signals only were not significantly different from chance in either the intermediate (*t*(19) = −2.035, *n.s.*, FDR corrected) or bottom-up conditions (*t*(19) = −5.685, *n.s.*, FDR corrected) in which the same grid-shaped stimulus-configuration as in the top-down condition was used. In accordance with the top-down condition results, EEG signals yielded significantly higher decoding accuracies than EOG signals in both intermediate (*t*(19) = 9.181, *p* < 0.0001, FDR corrected) and bottom-up conditions (*t*(19) = 10.967, *p* < 0.0001, FDR corrected). The decoding accuracies using CCA were significantly higher than chance in both the intermediate (*t*(19) = −12.2817, *p* < 0.0001, FDR corrected) and bottom-up conditions (*t*(19) = −12.584, *p* < 0.0001, FDR corrected). Moreover, the decoding accuracies using CCA gradually increased from the top-down (19.23%) to the intermediate (35.04%) and further to the bottom-up condition (53.79%; *F*(2,57) = 68.463, *p* < 0.001). However, similarly to the top-down condition, LDA yielded significantly higher decoding accuracies than CCA in both the intermediate (*t*(19) = 6.453, *p* < 0.0001, FDR corrected) and bottom-up conditions (*t*(19) = 8.360, *p* < 0.0001, FDR corrected).

### Activation pattern and Granger-causal connectivity

Based on the activation patterns of neural sources that were obtained from analyzing the BMI classifier, the top-down SSVEP condition resulted in a distinctive topographical distribution of activation, which showed a higher degree of focus around the occipital electrodes. Conversely, the bottom-up SSVEP condition showed a more dispersed BMI feature activity (Fig. 3b). The intermediate SSVEP condition showed a moderately focalised BMI-feature scalp distribution in between that of the other two conditions. In addition, the maximum amplitudes of these activation-pattern maps systematically decreased from the bottom-up (averaged maximum, 0.281) to the intermediate (averaged maximum, 0.187), and finally to the top-down condition (averaged maximum 0.093; *F*(2,57) = 49.117, *p* < 0.001, see Fig. 3b). Furthermore, it is noteworthy that only the top-down SSVEP condition showed anterior prefrontal (i.e., Brodmann’s area (BA) 10) regularization over the occipital visual association region BA 18 (see the direction of arrows in Fig. 3c) when Granger causality analysis^44^ was conducted. The intermediate and bottom-up conditions demonstrated the opposite directional information flow: from the occipital to the frontal region. In addition, there was strong crosstalk between the left and right anterior prefrontal areas (BA 10L and BA 10R), particularly in the top-down SSVEP condition. All of these directed functional connectivity measures were reliably confirmed by additional observations using time-reversed data^45^,^46^ (see the Supplementary information). However, neither the intermediate nor the bottom-up SSVEP conditions showed true directed functional connectivity because their time-reversal results in Granger causality were not significantly different from those of the original data, which is indicative of spurious causal relations, such as a volume conduction effect^45^,^46^.

## Discussion

This study proposes a novel approach to decode a user’s multi-class intention in the context of SSVEP-BMI. It is noteworthy that EOG signals could not explain the observed effects. Although the decoding accuracy of the grid-shaped top-down SSVEP paradigm can still be improved as compared with other SSVEP-based BMIs (Table 2), it has shown promising first results toward future BMI technology able to recognize complex multi-class intention without gaze-shifting (i.e., covertly). The proposed grid-shaped SSVEP-based BMI technique is substantially different from classical SSVEP-BMI paradigms in the following aspects.

First, when inspecting the neural activation patterns obtained from analyzing the BMI classifier^47^, the discriminant neural correlates of the top-down SSVEP paradigm are more localized around the occipital area (Fig. 3b), suggesting more focused visuospatial attention processing. Previous studies consistently reported that anticipatory biasing of visuospatial attention by task-strategy focalizes brain activity on the occipital area^48^,^49^, which may reflect preparatory top-down processing in early information processing stages in occipital visual areas^50^,^51^. Since participants had to allocate more selective visuospatial attention (i.e., increased top-down control) to the specific flickering lines corresponding to the conceived letter and simultaneously inhibit information processing for the task-irrelevant flickering lines, the activated occipital visual area is more focalized during the top-down condition.

Second, strong communication between the left and right anterior prefrontal areas (i.e., BA 10L/R) is only observed during the top-down SSVEP condition, in which robust prefrontal cortical (PFC)-dependent top-down control over the occipital visual association area BA 18 is detected by Granger causality analysis (Fig. 3c). It is likely that prefrontal top-down processing influences low-level early visual processing stages. BA 10 is the anterior prefrontal area, which is involved in the cognitive processing of attention^52^, semantic monitoring^53^, working memory^54^, rule learning^55^, decision-making^56^, inhibitory control^57^, and the compromised functioning of top-down regulation^58^. On the other hand, BA 18 is affiliated with the extrastriate visual cortex, where the visual information from the primary visual cortex (specifically visuospatial selective attention^59^) is further processed. Since attention enhances extrastriate neuronal responses to a stimulus at one spatial location in the visual field^60^, prefrontal top-down influence when placing one’s attention selectively on a corresponding line combination among six flickering lines might downregulate BA 18 activity. In contrast, the intermediate and bottom-up SSVEP conditions show the opposite direction of information flow: from the occipital to the frontal cortex. These observations reflect the difference between the top-down SSVEP condition and the passively induced bottom-up processing of the typical occipital SSVEP paradigm that is physically driven by externally flickering visual stimuli. However, this is not the case in the top-down SSVEP condition, because the stimulus-driven (bottom-up) properties of the early visual cortices are common across the six class-stimuli. Instead, higher-order cognitive processing stages such as those in prefrontal regions might be involved as a control centre for selectively attending to a specific line composite structure across this similar stimulus, based on the intentionally conceived letter. It has been consistently reported that stimulus-driven attention processes are neuroanatomically dissociable from the intentionally driven top-down processes of visuospatial selective attention^61^. Therefore, our findings provide neurophysiological evidence that the present paradigm requires top-down control (e.g., selective attention processing) when users intend to conceive of letters for task performance. The interaction between PFC-dependent top-down control and occipital bottom-up processing might be anatomically accomplished through the fronto-occipital fasciculi, providing reciprocal connections between the PFC and the occipital cortices^62^. This notion is in line with reports that slow oscillatory activity is associated with long-distance neural network function^63^, since we observed such directed fronto-occipital functional connectivity in the theta band. The extensive and reciprocal connections between the PFC and all other brain regions provide a neuroanatomical substrate for the role of the PFC in controlling diverse cognitive processes^64^.

In addition, it is noteworthy that CCA, which is commonly used for decoding SSVEP signals^26^,^36^, showed lower performance for the proposed top-down SSVEP condition compared to rLDA (Fig. 3a; the intermediate SSVEP condition consistently shows moderate CCA decoding accuracy between top-down and bottom-up SSVEP conditions). The CCA approach has an advantage for classification when the SSVEPs are physically driven by the repetitive presentation of a visual stimulus at a given flickering frequency. As shown in Fig. 3a, the decoding accuracies using CCA gradually increased from the top-down to bottom-up conditions. These different characteristics of the typical SSVEP-BMI classification technique also provide additional evidence that the proposed top-down SSVEP paradigm is quite distinct from the classical bottom-up SSVEP paradigm. The extracted features of the top-down SSVEP paradigm seem to be highly associated with higher-order top-down attributes as opposed to the bottom-up properties of classical SSVEP stimuli.

Taken together, the proposed grid-shaped top-down SSVEP paradigm provides potential to expand the gaze-shift-free intention recognition BMI repertoire. This technique has further advantages. First, the SSVEP-based BMI conveys very precise information^14^ and has a high ITR, requiring the fewest number of recording electrodes possible^15^,^65^, which enables this technology to be easily wearable. Indeed, since only three occipital electrodes can efficiently represent discriminative features in the top-down SSVEP BMI paradigm, this system may feasibly be used as a wearable BCI device. In addition, users do not need to explicitly move their eyes; as shown above, communication is more successfully achieved by decoding EEG than EOG. Therefore, the extracted physiological signature of this technique reflects genuine brain activity, not eye movement. Some of the recently proposed SSVEP-mediated BMIs inevitably require eye movements for attributing focal attention to a flickering symbol^25^,^26^,^27^,^28^. This may alternatively be detectable by an eye tracker; however, the present technique avoids the necessity of macroscopic eye movements by using a small-sized grid-shaped line array corresponding approximately to the foveal visual angle.

Therefore, the proposed top-down SSVEP paradigm may become a potent future technology for intentional control of a multi-class BMI. However, this paradigm needs further improvement in subsequent studies. The limitations of this study are as follows. First, the decoding accuracy and ITR of the grid-shaped top-down SSVEP paradigm have room for improvement. For example, by using a shorter epoch size and selecting optimal electrodes for feature-discrimination, the ITR and accuracy, respectively, could be further enhanced. Second, as users must look at flickering stimuli in order to generate such SSVEPs, they inevitably experience eye-fatigue^24^. However, this can be overcome using high-frequency SSVEP technology^29^,^66^. The high-frequency flickering produces much less visual fatigue than that at lower frequencies^67^,^68^, making the SSVEP-based BCI a more comfortable and stable system^67^. However, it still remains to be further studied for practical BMI application. Third, since the grid-shaped SSVEP technique is currently implemented using the Korean letter system, a study in which it is transferred to other languages is required for its versatile usage. Fourth, the number of letters that can be formed through grid-shaped stimulation is currently limited. Further refinement, by adding more rows and columns to the grid-shaped line array (which enables decoding of a larger set of letter-like shapes), would, in principle, allow the technology to be used for a variety of accurate mind-reading applications ranging from communication and neuro-rehabilitation to general consumer electronics. Unlike most previous studies in SSVEP-based BMIs, which generally use fewer than four flickering frequencies that may even be located in different parts in the visual field^15^ and thus require ocular movements, the present top-down SSVEP uses a highly compact stimulus presentation size. Therefore, our novel paradigm provides multi-class intention decoding, which could in principle decode a combinatorial number of letter-like configurations – a technique that may ultimately become useful for both patients and healthy user groups. In particular, our gaze-independent SSVEP-BMI paradigm will benefit for paralyzed end-users suffering from amyotrophic lateral sclerosis (ALS) or complete locked-in syndrome.

As compared with our findings of decoding covert attentional shift to subsets of external stimuli, there are other recent studies decoding visuospatial attention directly from the intrinsically driven brain activity patterns evoked by attention^69^,^70^,^71^,^72^,^73^. As our study demonstrates the possibility of seamlessly decoding human top-down processes, if a further refined grid-shaped line array is developed and combined with a more elaborated classification method, a recent study^74^ provides advances in extending previous studies of decoding visuospatial attention^69^,^70^,^71^,^72^,^73^, which were limited to decoding up to four discrete locations/classes. The authors found a neuronal signature of direct two-dimensional access to the spatial location of covert attention in macaque prefrontal cortex^74^. Together, these efforts in both externally triggered and internally driven manners may cooperatively open new perspectives to decoding technology in a multi-class and continuous mental representation space.

## Methods

### Participants

Twenty healthy subjects (10 female; mean age 25.7 y) participated in this study, which was conducted in accordance with the ethical guidelines established by the Institutional Review Board of Korea University and the Declaration of Helsinki (World Medical Association, 2013). All experimental protocols were approved by the Institutional Review Board of Korea University (No. KU-IRB-13–43-A-2). Participants provided informed consent prior to the start of the experiment. All had normal or corrected-to-normal vision.

### SSVEP-inducing grid-shaped line array

**Figure 1:**
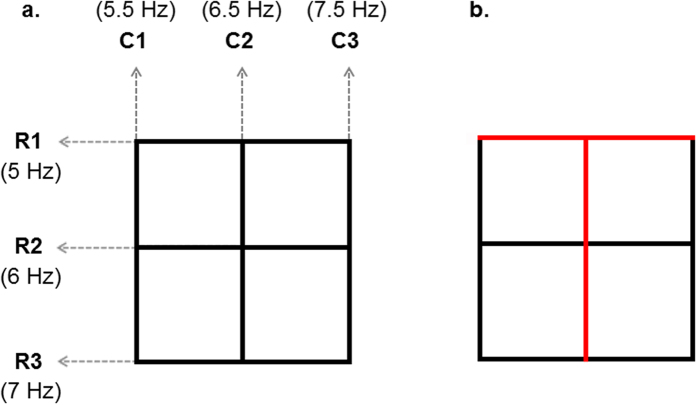
A schematic design of an SSVEP-inducing grid-shaped line array. (**a**) Grid-shaped line array consisting of three rows (R1, R2, and R3) and three columns (C1, C2, and C3) of individually flickering lines. (**b**) Example of an attended flickering line composite (in red) when a participant pays particular attention to the Korean letter ‘⊤’ while looking at the flickering grid-shaped line array.

In order to present a mentally generated letter within the participant’s restricted visual angle to evoke the corresponding SSVEP, a 6 cm × 6 cm grid-shaped line array was designed (see Fig. 1 and Supplementary video clip 1). In this array, three rows (R1, R2, and R3 in Fig. 1a) and three columns (C1, C2, and C3 in Fig. 1a) of lines with mean luminance of 136.26 cd/m^2^ were lit on a black monitor (Full HD LED 27-in., S27B550, Samsung, Seoul, Korea). Each line had a width of 6 mm and the distance between two adjacent rows or columns was 1 cm. This grid-shaped line array was within the visual angle of 6.4° at a distance of 65 cm^75^, falling on the retinal region centred at the fovea (the most sensitive portion of the retina) without macroscopic eye-movement. In order to generate individual SSVEPs based on each flickering line, each row and column had an individual flickering frequency ranging from 5 to 7.5 Hz (see Table 1 and Fig. 1a), which have been shown to be effective frequencies for inducing SSVEPs in humans^76^,^77^. The frequencies were assigned randomly to each line in the overall array. A sampled sinusoidal stimulation method^26^ was used to implement visual stimulus presentation on the LED screen for eliciting SSVEP responses.

The underlying idea of decoding a participant’s top-down modulated responses to external stimulation by means of SSVEPs induced by this grid-shaped line array was as follows. When a participant paid attention to a subset of flickering lines representing the shape of a letter or symbol, the corresponding frequencies of those lines were expected to be detected as the dominant SSVEP features. The frequencies driving the SSVEP signals could be analyzed using a pattern recognition algorithm (detailed in the *Analysis method* section) and decoded to identify the symbol shape intended by the participant. The experiment consisted of three conditions: top-down, intermediate, and bottom-up SSVEP. In order to make all the conditions’ results comparable, the grid-shaped line array structure discussed above was used in all three conditions. In order to rule out luminance effects across the top-down and bottom-up conditions, overall stimulus luminance was maintained as closely as possible. However, in the bottom-up condition, all lines belonging to the same object had the same flickering frequency (5, 5.5, 6, 6.5, 7, or 7.5 Hz; see Supplementary video clip 3). In an intermediate SSVEP condition between the top-down and bottom-up conditions, the luminance of task-irrelevant flickering lines was reduced by 1/10 of the original (see Supplementary video clip 2). Thus, the task-relevant lines were much brighter, in order to facilitate the participant’s recognition of the appropriate symbol. Each condition comprised 4 blocks with a short break in between; each block included 60 trials. In each block, each of 6 Korean letters (phonemes) was presented 10 times in a random order. The inter-trial intervals ranged from 1000 ms to 1500 ms, centred at 1250 ms. After a 1 s auditory cue pronouncing the Korean letter to which a participant was required to attend, and a subsequent 500 ms buffer period, the grid-shaped line array was presented for 5 s. During these 5 s, the participant was expected to focus his/her attention on the instructed combination among all 6 flickering lines. In the bottom-up SSVEP condition, an auditory cue (an analogue instruction sound) was presented in a random order to control for possible linguistic region activation induced by the spoken instruction itself. In other words, a randomly selected auditory cue from the six Korean letters was presented during the bottom-up SSVEP condition, in which all six line segments in the grid-shaped line array flickered at the same frequency as a single object. Using the same stimulus object (i.e., the grid-shaped line array), we accomplish a classical SSVEP paradigm (i.e., a single flickering object is presented to evoke SSVEPs)^22^,^32^. Through this bottom-up experimental design, we tried to minimize any confounding effects possibly introduced by differing stimulus size and shape, and thus to make its results comparable with the observations from both the top-down and intermediate conditions.

### EEG acquisition

The EEG was measured using a BrainAmp DC amplifier (Brain Products, Germany) with 32 Ag/AgCl electrodes in an actiCAP (Brain Products, Germany) in accordance with the international 10–10 system. An electrode was placed on the tip of the nose as reference, and a ground electrode was placed at electrode AFz. Eye movement activity was monitored with an EOG electrode placed sub-orbitally to the left eye, and vertical and horizontal electro-ocular activity was computed using two pairs of electrodes placed vertically and horizontally with respect to both eyes (i.e., Fp1 and EOG for the vertical EOG, F7 and F8 for the horizontal EOG). The EOG was used to track gaze-shifts. Electrode impedances were maintained below 5 kΩ prior to data acquisition. The EEG was recorded at 500 Hz.

### Analysis methods

**Figure 2:**
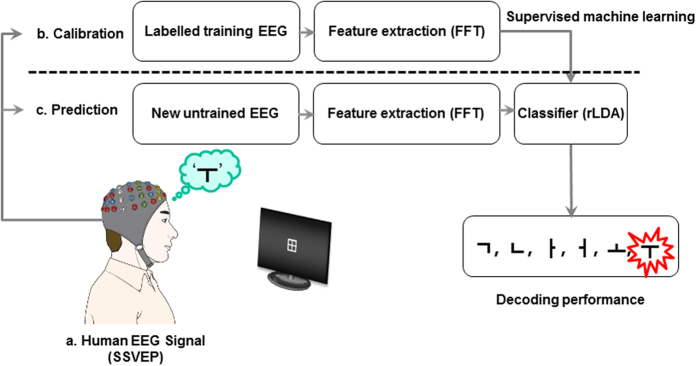
Schema for the workflow of a top-down SSVEP paradigm for multi-class decoding BMI technology. (**a**) EEG recording, (**b**) Calibration by supervised machine learning from training EEG trials, and (**c**) Decoding process on test EEG trials to correctly predict participant perception. When a participant conceives the letter ‘⊤’, the corresponding flickering line composite on the grid-shaped array is subsequently attended and a classification algorithm using feature extraction (i.e., rLDA), calibrated through supervised machine learning, enables the successful decoding of the originally conceived letter ‘⊤’. FFT stands for ‘fast Fourier transform’ and rLDA represents ‘regularized linear discriminant analysis’.

A supervised machine learning method^78^ was trained during a calibration phase using labelled training EEG trials. The task of the multi-class classifier is to extract a task-relevant signal from the EEG, which is used to assign the recorded samples to a given stimulus category^7^. In the present study, rLDA with shrinkage^8^,^40^ was applied to channel-wise computed power spectral densities. The power spectral densities of all channels were obtained by fast Fourier transform (FFT) using the Berlin Brain-Computer Interface (BBCI) toolbox^79^. Classical LDA is optimal in the sense that it minimizes the risk of misclassification for new samples drawn from known Gaussian distributions^80^. Particularly, rLDA is a powerful and robust machine learning technique that yields excellent results for single-trial event-related potential classification, which are superior to classical LDA when the ratio of features to trials is low^8^,^40^,^42^. The power spectral densities ranging from 5 Hz to 13.5 Hz (in 0.5 Hz increments) of the 5 s EEG signals were used for feature extraction. This frequency range includes the stimulus flickering frequencies along with the sum of letter-corresponding combination frequencies. After fixing the parameters of the rLDA on the training data, the resulting calibrated classifier was used for out-of-sample prediction, i.e., novel unseen EEG trials could be decoded (Fig. 2). We performed 4-fold chronological cross-validation^40^ to obtain out-of-sample classification performance; we thus designated 180 trials for training and the remaining 60 trials for testing, out of all 40 trials per stimulus per participant (i.e., 240 trials including all stimuli per participant). First, all trials were chronologically split into 180 and 60 trials. During the cross-validation process, model (hyper-) parameters were chosen using the 180 trials and then the remaining 60 trials were tested using the trained model. This procedure was iterated 4 times to provide different combinations of training and test data sets and the resulting decoding accuracies were averaged. This decoding procedure was performed separately for each participant. The decoding accuracy was computed based on the signals of all 30 electrodes. In order to compare this result with the accuracy of the EEG signals from the three occipital electrodes (i.e., Oz, O1, and O2), the decoding accuracy of these three channels was also computed. The decoded signals were evaluated in terms of whether the information encoded in the set of attended flickering lines could be successfully reconstructed, i.e., whether the Korean letter that the participant was requested to conceive was correctly decoded. For the six-class classification, the rates of successful classification of the test data were compared across models for evaluating the decoding performance. In addition, rLDA-based classification was also performed using the EOG signals only, in order to compare EOG decoding accuracy with that based on EEG signals. We also conducted CCA^36^,^81^ on the same dataset to compare with the classification efficiency of rLDA. CCA is a multivariable statistical method to measure the linear correlation relationship between two sets of variables. Since CCA has been most frequently and successfully used in typical bottom-up SSVEP-based BMIs^26^,^36^, the results of this analysis would provide a solid comparison between classical bottom-up and the proposed top-down SSVEP paradigms in this work. In order to statistically examine whether the decoding accuracies were significantly different across the following models, the accuracies were analyzed with a repeated-measures analysis of variance (ANOVA) with one within-subjects factor: labeled ‘classification model’ (rLDA of EEG, randomly-shuffled rLDA of EEG, rLDA of EOG, and CCA of EEG). When necessary, the Greenhouse–Geisser correction was used. If statistical significance was observed, two-tailed paired *t*-tests were performed as post hoc pairwise comparisons: (1) rLDA of EEG vs. randomly-shuffled rLDA of EEG, (2) rLDA of EEG vs. rLDA of EOG, (3) rLDA of EEG vs. CCA of EEG, (4) rLDA of EOG vs. randomly-shuffled rLDA of EEG, (5) CCA of EEG vs. randomly-shuffled rLDA of EEG, and (6) rLDA of EOG vs. CCA of EEG. The randomly shuffled rLDA decoding accuracies were computed on the same training data but with randomly shuffled labels; thus its decoding accuracy represented the chance performance of the six-class classification. In addition, a one-way ANOVA was applied for testing any effects across the three experimental conditions. A FDR of *q* < 0.2^82^ was used to correct for multiple comparisons, since *q*-values between 0.1 and 0.2 after FDR correction are known to be acceptable for this purpose^83^. All analyses were performed using MATLAB (ver. R2015b, MathWorks, USA) or SPSS Statistics (ver. 22, IBM, USA).

**Figure 3:**
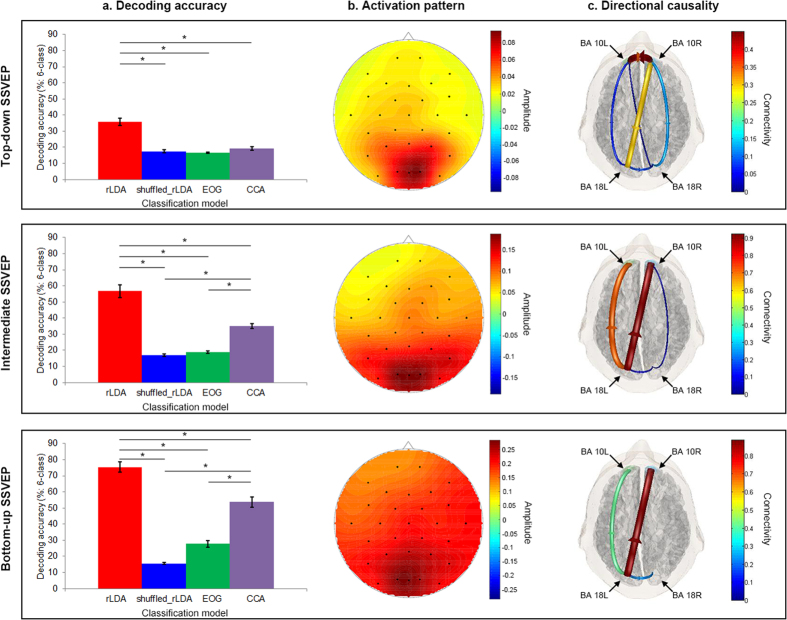
Decoding accuracies, topographical activation patterns, and Granger causality. (**a**) Comparison of decoding accuracies across EEG rLDA (red bars), randomly shuffled EEG rLDA (blue bars), EOG rLDA (green bars), and EEG CCA (purple bars). The accuracy of random selection (i.e., 17.44%) is computed by the randomly shuffled rLDA method (blue bars), and error bars indicate ±1 standard error of the mean across participants. *p < 0.0001, two-tailed paired *t*-tests were conducted across these comparisons, and multiple comparisons were corrected using the false discovery rate (FDR). (**b**) Topographical activation patterns of neural bases for BMI features are computed for the top-down, intermediate, and bottom-up SSVEP paradigms. The degree of differences in normalized classifier weights is depicted in a coloured scale; large weights are strongly related to the task condition. Note the gradual decrease in the amplitude of activation patterns from the top-down to bottom-up SSVEP paradigms. Black dots represent the electrode positions. (**c**) Directional information flows between BA 10 (anterior prefrontal cortex) and BA 18 (occipital visual association cortex) by Granger causality analysis. Using the estimated time courses of the two ROIs, the directed transfer function (DTF) analysis identified directional information flow across cortical sources. Note the directions of the arrows that indicate the direction of information flow. The coloured scale and the line thickness represent the degree of directed functional connectivity (ranging from 0 to 1). Only statistically significant directed functional connectivity patterns are illustrated. The view of the topography is from the vertex, with the nasion at the top of the image.

In order to gain better understanding of the classifier with respect to the neurophysiological basis of the extracted task-relevant signal, an ‘activation pattern’ approach^47^ was adopted in the present study (see Fig. 3b). The learned parameters of linear classifiers such as rLDA and CCA (i.e., their weight vectors) cannot be interpreted with respect to the origin of the signal of interest because the parameters of the models are a function of the task-relevant signal and the task-uninformative signals (i.e., noise signals)^8^,^40^,^47^. Therefore, in order to visualize how the extracted signal is encoded in the features that are used by the classifier, a so-called ‘activation pattern’ has to be computed^47^,^84^. Assuming that the task-relevant and task-uninformative signals are uncorrelated, the activation pattern is given by the covariance between the classifier output and the time-course of individual features^47^. In order to make the activation patterns comparable across conditions, we computed their correlation instead of their covariance. We estimated an activation pattern involving all scalp electrodes by computing the correlation between the continuous trial-wise classifier output and the time-course of spectral features (i.e., trial-wise spectral power in the chosen frequency bins) from all channels. In order to arrive at a single activation pattern for each condition that could be visualized as a scalp map, the activation patterns were averaged across frequency bins and participants (see Fig. 3b).

The spatiotemporal distribution of brain activity and network behaviour provide significant psychophysiological information. Given that it is important to image functional connectivity to understand brain function^85^,^86^, Granger causality^44^ analysis was also conducted in this study. In particular, the directed transfer function (DTF) has been developed to describe causality among an arbitrary number of signals^87^. Granger causality analysis has shown potential for non-invasively delineating brain network connectivity^88^. Using the eConnectome software^86^, functional connectivity was mapped for each experimental condition. Granger causality was investigated in the frequency band from 5 Hz to 8 Hz, which includes the range of the stimulus-flickering frequency. The eConnectome software enables cortical source imaging and subsequent connectivity analysis of cortical source activity. To estimate the source-level cortical activity, a source-localization software, LORETA, or low-resolution electromagnetic tomography, (version 20151222, The KEY Institute for Brain-Mind Research, Switzerland) was employed herein^89^. LORETA is one method that estimates the electric neural generators and computes images of neural activity from EEG data. eLORETA is tested under computer-controlled conditions, using a realistic head model, with 7002 cortical voxels^90^. We calculated eLORETA images during task performance in the time frame from 0 to 5 s. Based on the most pronounced cortical activity estimated by the LORETA software, two regions of interest (ROIs) were bilaterally selected (i.e., BA 10 and 18) to map directional connectivity. Source waveforms at the two ROIs were estimated and the DTF analysis showed directional information flow across sources (see Fig. 3c). Statistical assessment of the connectivity was performed using the surrogate approach (1000 surrogates, *p* < 0.05). In order to avoid spurious causal relations, we conducted the same Granger causality analysis using time-reversed data^45^,^46^. That is, the reversed temporal order of all data points in the same EEG dataset was used to double-check the robustness of the inferred Granger-causal connectivity measures.

## Additional Information

**How to cite this article**: Min, B.-K. *et al*. Decoding of top-down cognitive processing for SSVEP-controlled BMI. *Sci. Rep.* **6**, 36267; doi: 10.1038/srep36267 (2016).

**Publisher’s note:** Springer Nature remains neutral with regard to jurisdictional claims in published maps and institutional affiliations.

## Supplementary Material

Supplementary Information

Supplementary Information

Supplementary Information

Supplementary Information

Supplementary Information

## Figures and Tables

**Table 1:** Letter-shaped stimuli and the flickering frequencies of their corresponding line composites.

Index	Stimulus	Horizontal line frequency	Vertical line frequency
1	**┐**	5 Hz (R1 in Fig. 1a)	7.5 Hz (C3 in Fig. 1a)
2	**└**	7 Hz (R3 in Fig. 1a)	5.5 Hz (C1 in Fig. 1a)
3	**⊥**	7 Hz (R3 in Fig. 1a)	6.5 Hz (C2 in Fig. 1a)
4	⊤	5 Hz (R1 in Fig. 1a)	6.5 Hz (C2 in Fig. 1a)
5	**⊢**	6 Hz (R2 in Fig. 1a)	5.5 Hz (C1 in Fig. 1a)
6	**⊣**	6 Hz (R2 in Fig. 1a)	7.5 Hz (C3 in Fig. 1a)

**Table 2:** Comparison of characteristics of recent SSVEP-based BMI systems.

Study	Stimuli	Task	Number of Electrodes	Number of Commands	Accuracy	ITR	Gaze-shift
The present study	Line-grid	Visual attention (N = 20)	3	6*	42.5% (20.0–63.3%)	3.2 (0.1–9.4)	No
Kwak *et al*.^17^	LED	Exoskeleton control (N = 11)	8	5	91.3% (81.4–98.6%)	32.9 (19.6–51.0)	Yes
Chen *et al*.^91^	Characters	Visual attention (N = 12)	9	40	91.0% (77.0–99.5%)	267.0 (199.8–315.0)	Yes
Chen *et al*.^26^	Characters	Visual attention (N = 10)	9	45	88.7% (73.3–98.9%)	61.0 (45.0–75.0)	Yes
Nakanishi *et al*.^92^	Rectangles	Visual attention (N = 13)	16	32	91.4% (84.4–98.4%)	166.9 (126.3–192.3)	Yes
Chen *et al*.^93^	Rectangles	Visual attention (N = 10)	64	8	93.8% (87.5–100%)	33.8 (28.1–40.0)	Yes
Bin *et al*.^36^	Rectangles	Visual attention (N = 12)	9	6	95.3% (83.3–100%)	58 (40–67)	Yes
Müller-Putz *et al*.^19^	LED	Prosthesis control (N = 4)	4	4	72.5% (44.0–88.0%)	19.7 (4.1–34.2)	Yes
Martinez *et al*.^94^	Checkerboard	Navigating game (N = 5)	6	4	96.5% (82.3–100%)	29.6 (17.0–38.7)	Yes

^*^More expandable, Information transfer rate (ITR) in bit/min, N: number of participants, LED: light-emitting diode, Gaze-shift: whether the visual angle of the stimulus range can eventually induce macroscopic gaze-shift.
