# Supplementary material for: Decoding of top-down cognitive processing for SSVEP-controlled BMI
Source: Sci Rep. 2016 Nov 3;6:36267. doi: 10.1038/srep36267 (PMC5093690; doi:10.1038/srep36267)
Supplement: Supplementary Information [file srep36267-s1.doc]

**Decoding of top-down cognitive processing for SSVEP-controlled BMI**

**Byoung-Kyong Min**^1^**,** **Sven Dähne**^2^**, Min-Hee Ahn**^1^**, Yung-Kyun Noh**^3^ **&** **Klaus-Robert Müller**^1,2^

^1^Department of Brain and Cognitive Engineering, Korea University, Seoul 02841, Republic of Korea, ^2^Machine Learning Group, Berlin Institute of Technology, Berlin 10587, Germany, and ^3^Department of Mechanical and Aerospace Engineering, Seoul National University, Seoul 08826, Republic of Korea

**SUPPLEMENTARY INFORMATION**

The following data are presented in a continuation of the results reported in the main text for further understanding of this study.

1. **Decoding accuracy**

To provide the decoding accuracies of all 20 participants, the following bar graphs (Fig. S1–S3) demonstrate individual decoding accuracies of the four different classification methods described in the main text: regularized LDA on EEG data, indicated by ‘rLDA’^1,2^; randomly-shuffled rLDA on EEG data, indicated by ‘rLDA(shuffled)’; regularized LDA on EOG data, indicated by ‘EOG’; and canonical correlation analysis on EEG data, indicated by ‘CCA’^3,4^. Error bars indicate ±1 standard error of the mean.


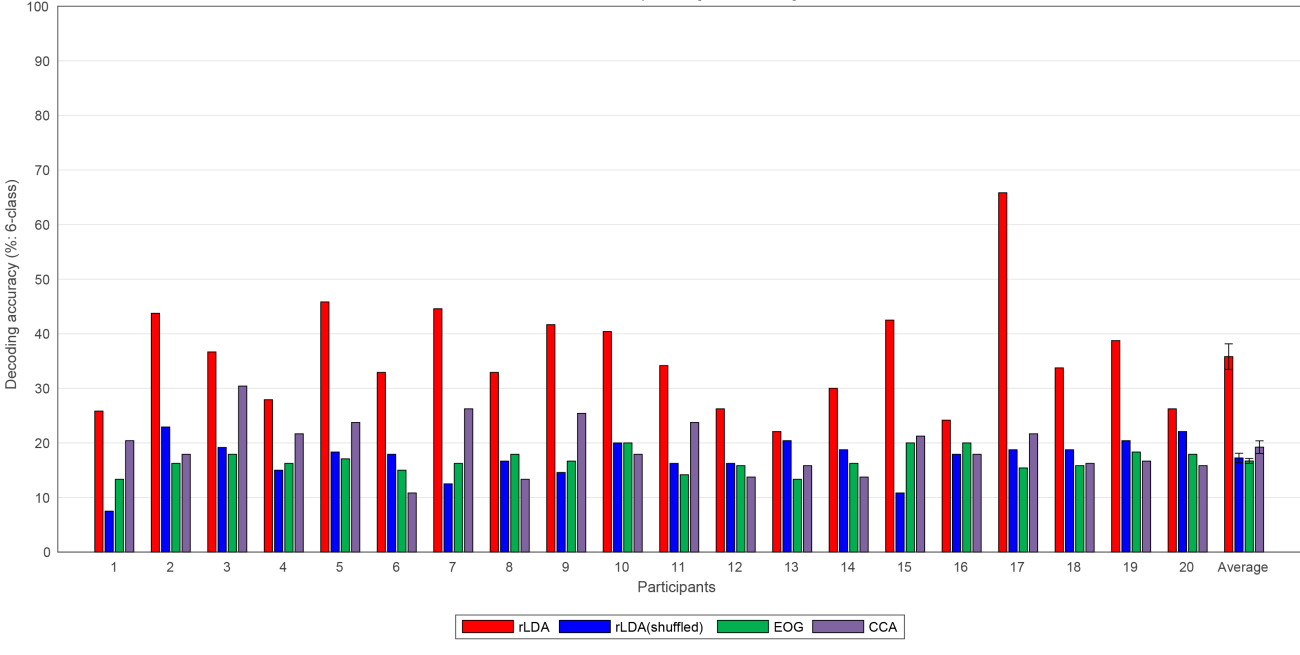


Supplementary Figure S1. Decoding accuracy of the top-down SSVEP condition


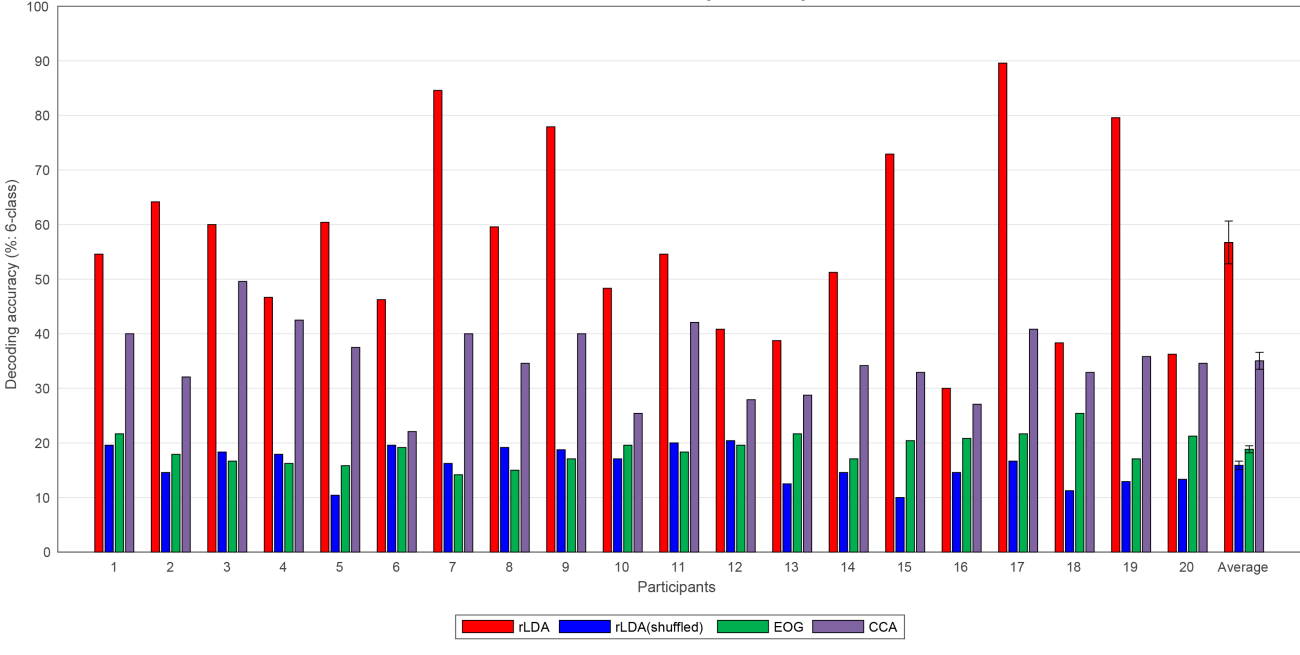


Supplementary Figure S2. Decoding accuracy of the intermediate SSVEP condition


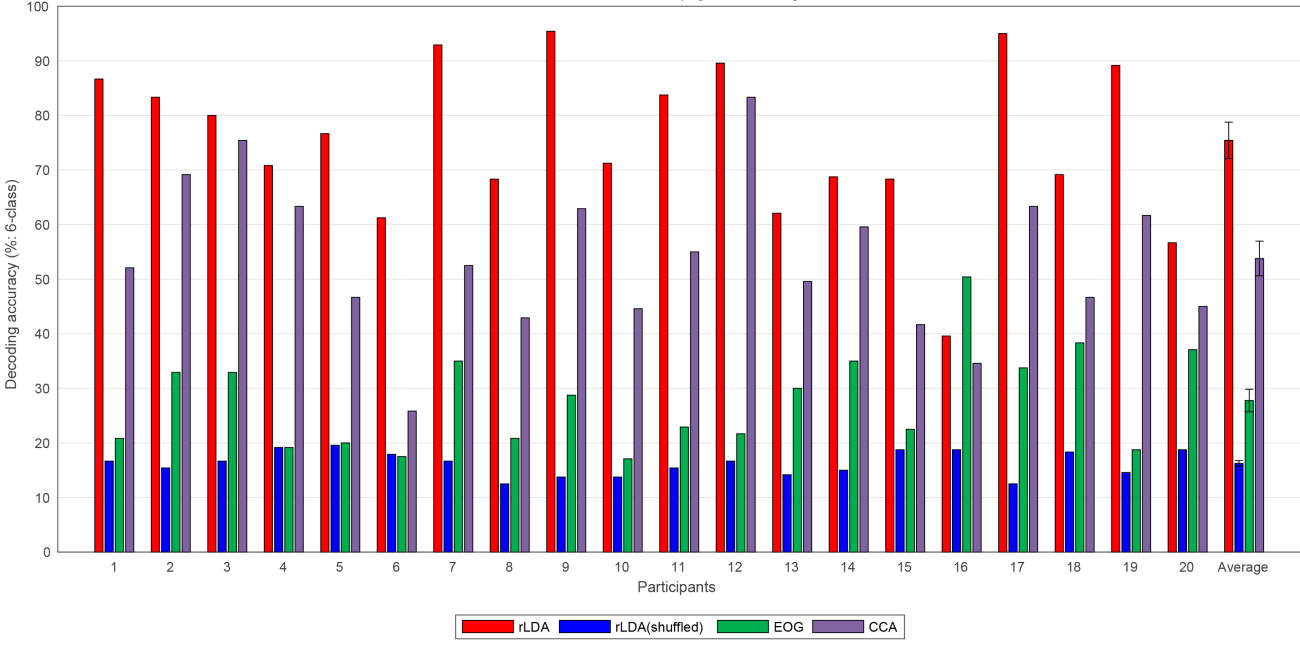


Supplementary Figure S3. Decoding accuracy of the bottom-up SSVEP condition

1. **Discriminability**

We computed the bi-serial correlation coefficient[^1^](#_ENREF_1) (*r^2^*) between the normalized power-spectral densities (ranging from 1 to 20 Hz) of the 5 s EEG signals and the stimulus type as shown in Fig. S4–S6. The power spectral densities of all channels were obtained by fast Fourier transform (FFT) using the BBCI toolbox^5^. Since the frequency range from 5 to 13.5 Hz includes the range of the stimulus-flickering frequency along with the sum of letter-corresponding combination frequencies, this frequency band showed a pronounced discriminability across the six different stimuli.

For the correlation analysis, a given stimulus type was encoded into a binary vector, which had a single entry per trial and which denoted whether the stimulus was the target stimulus for a trial or not. This binary vector was then correlated with the trial-wise power spectral densities of each frequency bin, separately for each recording channel. The result of this analysis is a single *r^2^*-value for every combination of subject, target stimulus, condition, frequency bin, and recording channel. Figures S4, S5, and S6 show the results for the three experimental conditions, respectively, averaged across subjects. Within each figure, the correlation values are shown as separate plots for each recording channel (see the respective channel label in the plot). Within each plot, the frequency bins are denoted on the x-axis and the *r^2^*-values for the six stimuli are shown as separate lines, respectively.

Note that the distinction between *r^2^*-values and the classifier pattern (see section *Analysis methods* in the manuscript) lies in the quantity that is correlated with the features; for the *r^2^*-values, the label signal is correlated and for the classifier patterns the output of the classifier is correlated. Furthermore, for the classifier patterns, we have aggregated the correlations across frequency bins and subjects.

It is noteworthy that the top-down SSVEP paradigm showed more focalised discriminability on the 3 occipital electrodes (i.e., Oz, O1, and O2) as compared to the bottom-up SSVEP paradigm that exhibited rather dispersed discriminability over the whole brain. In addition, the maximum amplitudes of these correlation coefficients systematically decreased from the bottom-up (greater than ±0.3) to intermediate (greater than ±0.1), and top-down conditions (greater than ±0.04).


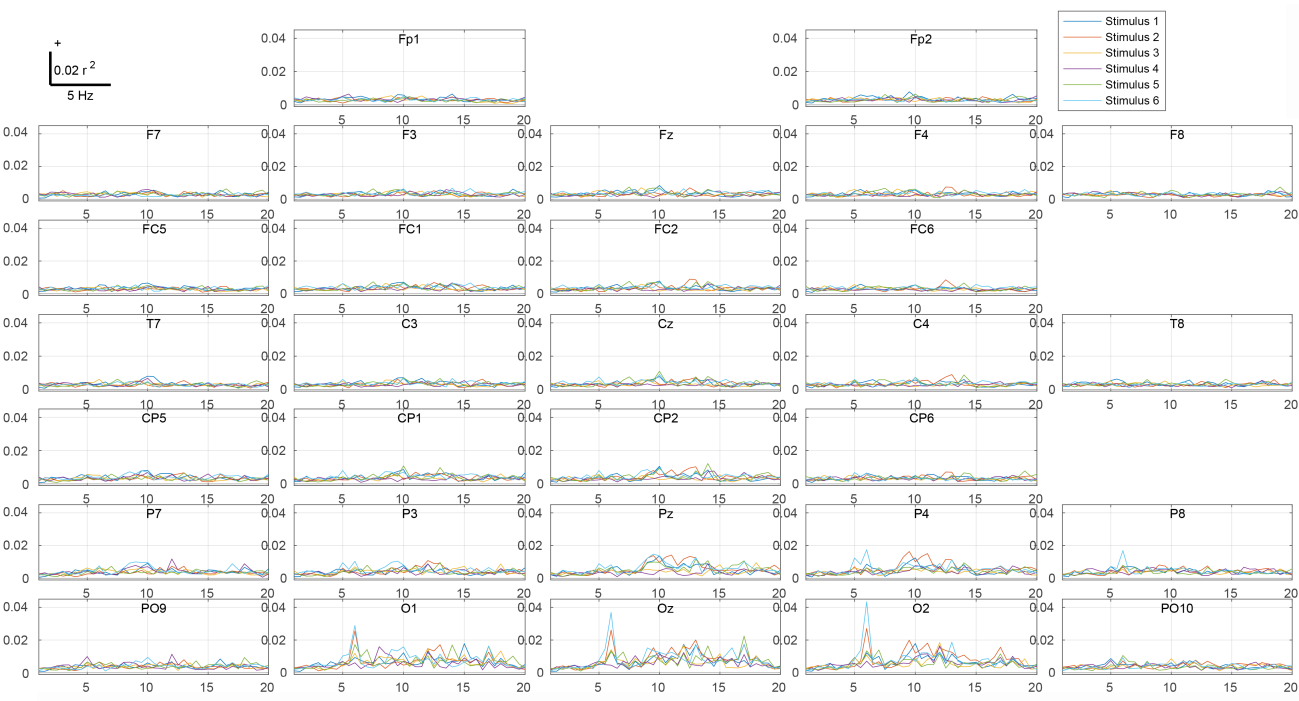


Supplementary Figure S4. Discriminability of the top-down SSVEP condition


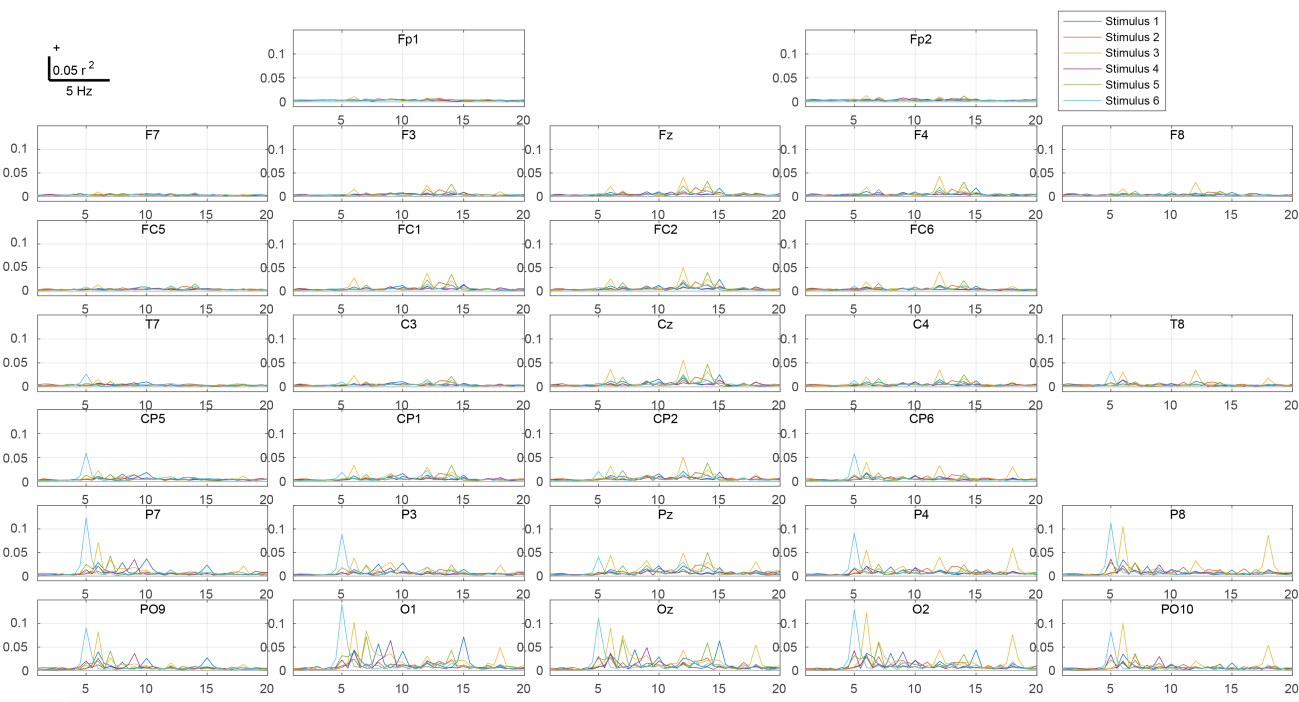


Supplementary Figure S5. Discriminability of the intermediate SSVEP condition


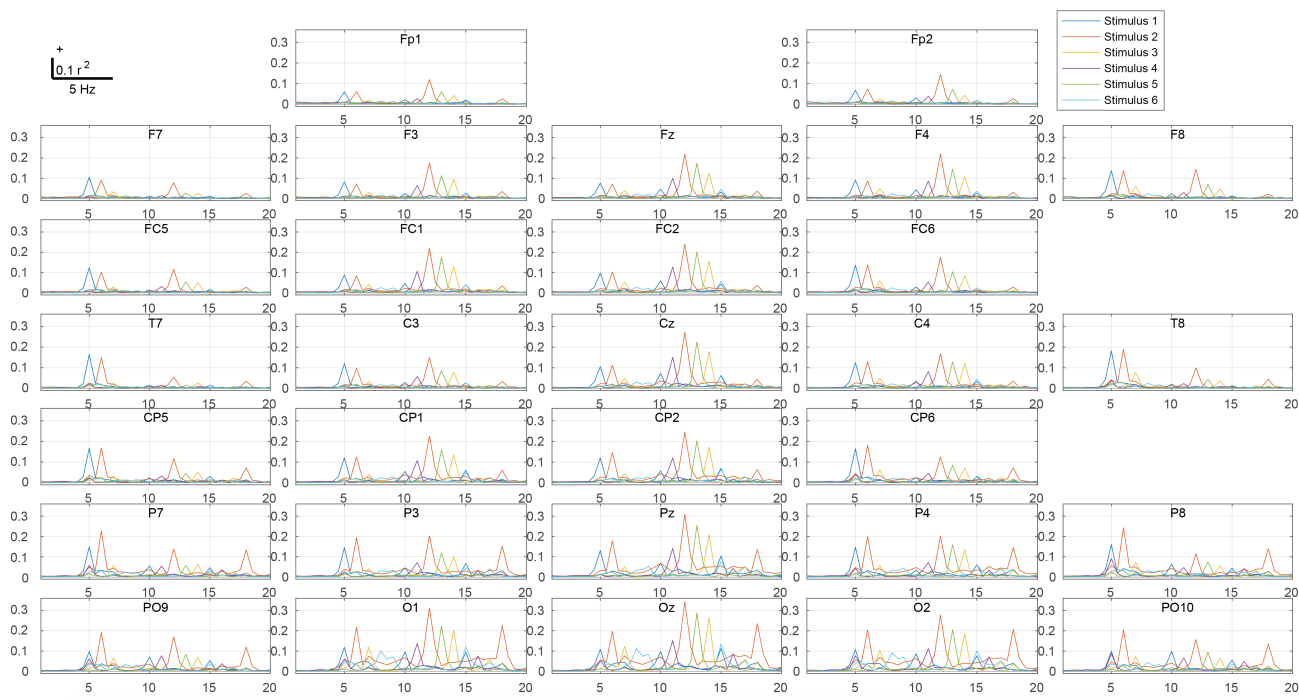


Supplementary Figure S6. Discriminability of the bottom-up SSVEP condition

1. **Power spectral density**

The power spectral densities ranging from 1 Hz to 20 Hz of the whole 5 s EEG signals are presented in Figs. S7–S9. The power spectral densities of all channels were obtained by fast Fourier transform (FFT) using the BBCI toolbox^5^. Note that the frequency range from 5 Hz to 13.5 Hz dominantly showed pronounced differences across the six stimulus types. This frequency band includes the range of the stimulus-flickering frequency as well as the sum of letter-corresponding combination frequencies.


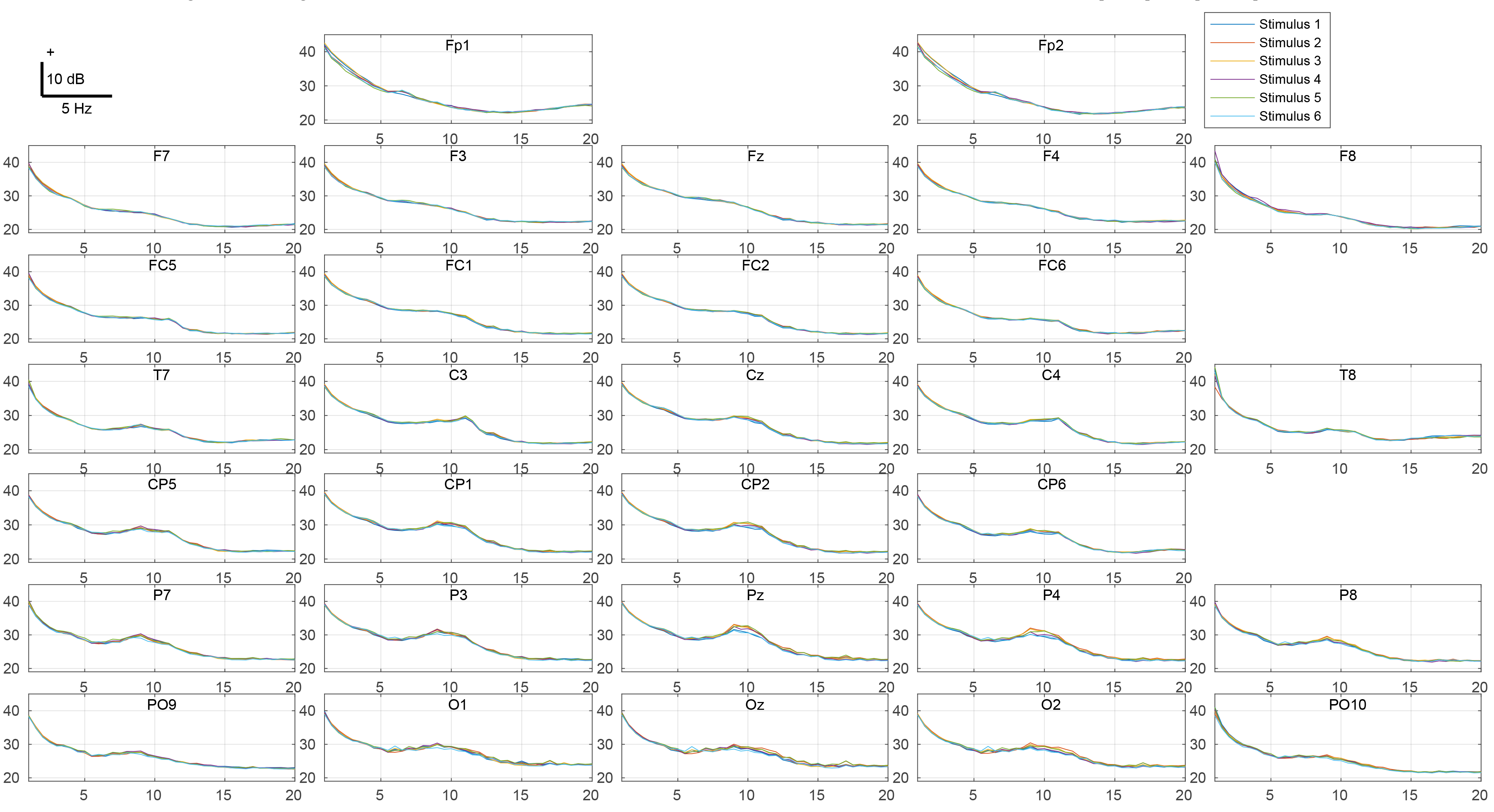


**Supplementary Figure S7. Power spectral density of the top-down SSVEP paradigm**


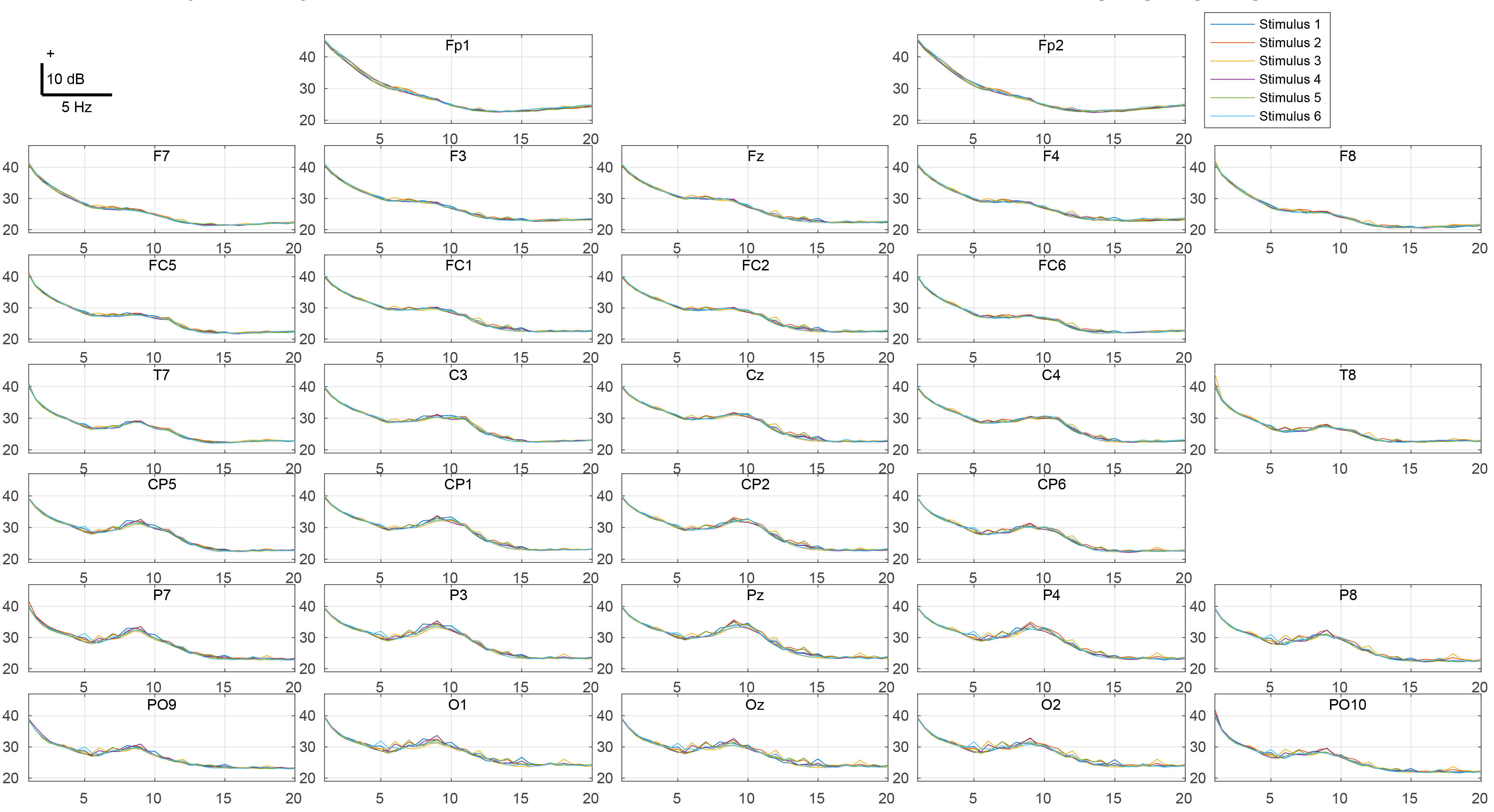


**Supplementary Figure S8. Power spectral density of the intermediate SSVEP paradigm**


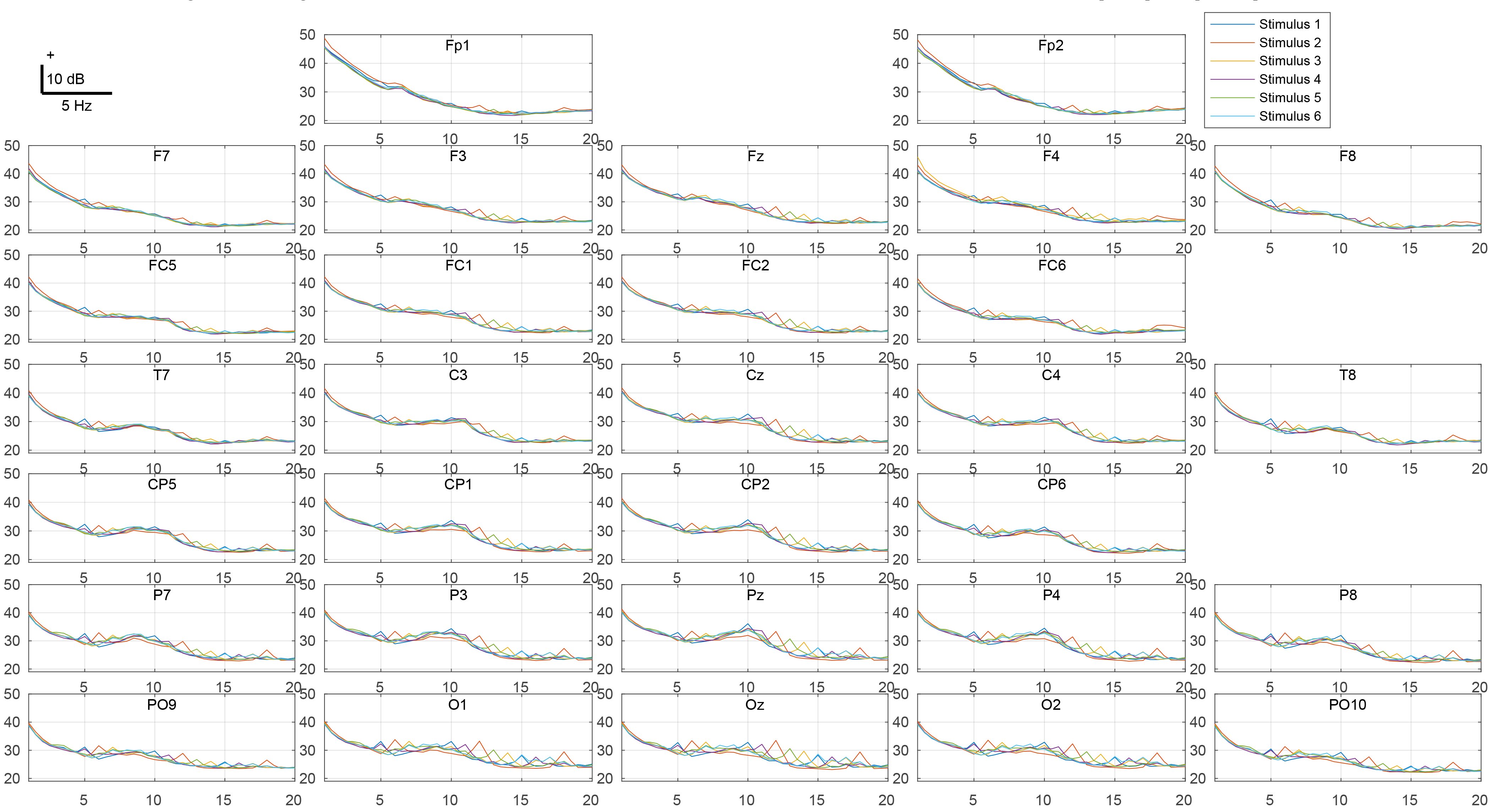


**Supplementary Figure S9. Power spectral density of the bottom-up SSVEP paradigm**

1. **Time-reversed test for the robustness of Granger causality**

In addition to the directional connectivity in the main text (Fig. 3c), the corresponding information flow images across 4 × 4 ROIs (*j*-to-*i* direction: *i*, *j* = 1 for BA 10L, 2 for BA 10R, 3 for BA 18L, and 4 for BA 18R) by Granger causality analysis are presented in Fig. S10. Using the estimated time courses of the 4 ROIs, DFT analysis identified directional information flow across cortical sources. As compared to both intermediate and bottom-up SSVEP paradigms, which yielded simple connectivity dominantly from BA 18L, the top-down SSVEP paradigm showed more directed functional connectivity across these ROIs, centred at the anterior prefrontal area. Particularly in the top-down SSVEP condition, there was strong crosstalk between the left and right anterior prefrontal areas (BA 10L and BA 10R), and the anterior prefrontal area (BA 10) dominantly downregulated the visual association area (BA 18) with strong causal connectivity. The colour-scaled directional arrows link two functionally connected ROIs when their Granger causality was statistically significant (i.e., *p* < 0.05). The colour-scaled pixels in the information flow map represent the degree of directed functional connectivity (ranging from 0 to 1).


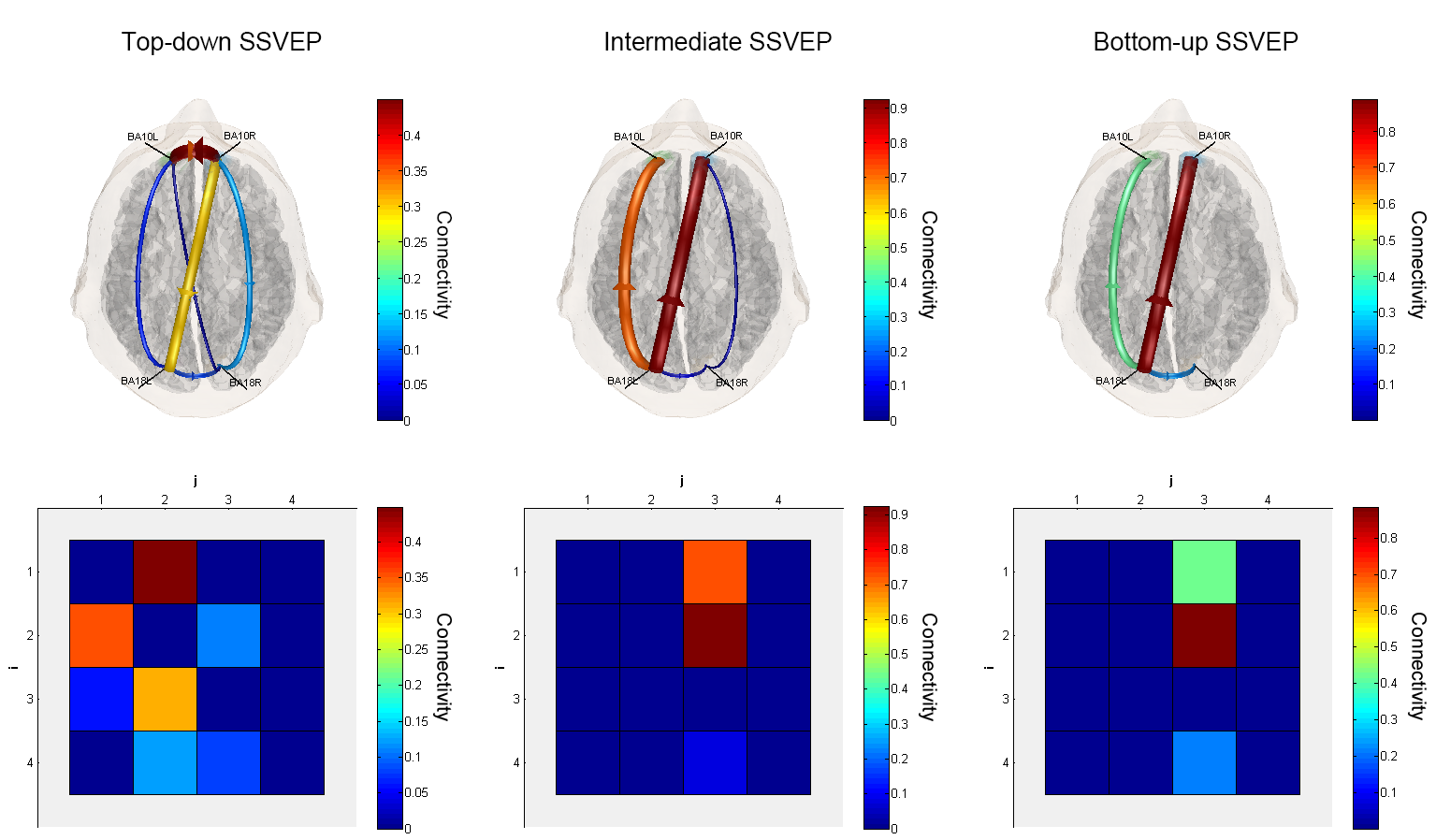


Supplementary Figure S10. Original Granger causal connectivity

In order to avoid spurious causal relations (for example, by volume conduction), we conducted the same Granger causality analysis using time-reversed data^6,7^ (Fig. S11). That is, the reversed temporal order of all data points in the same EEG dataset was used to check the robustness of the inferred Granger causal connectivity measures. We observed that only the top-down SSVEP condition showed a significant difference between the original and time-reversed Granger causal connectivity (F(1,11) = 7.539, *p* < 0.05). The other SSVEP conditions exhibited no significant differences (intermediate: F(1,11) = 0.109, *n.s.*; bottom-up: F(1,11) = 0.729, *n.s.*), which indicates that only the top-down SSVEP condition demonstrated true directed functional connectivity. The view of the topography is from the vertex, with the nasion at the top of the image.


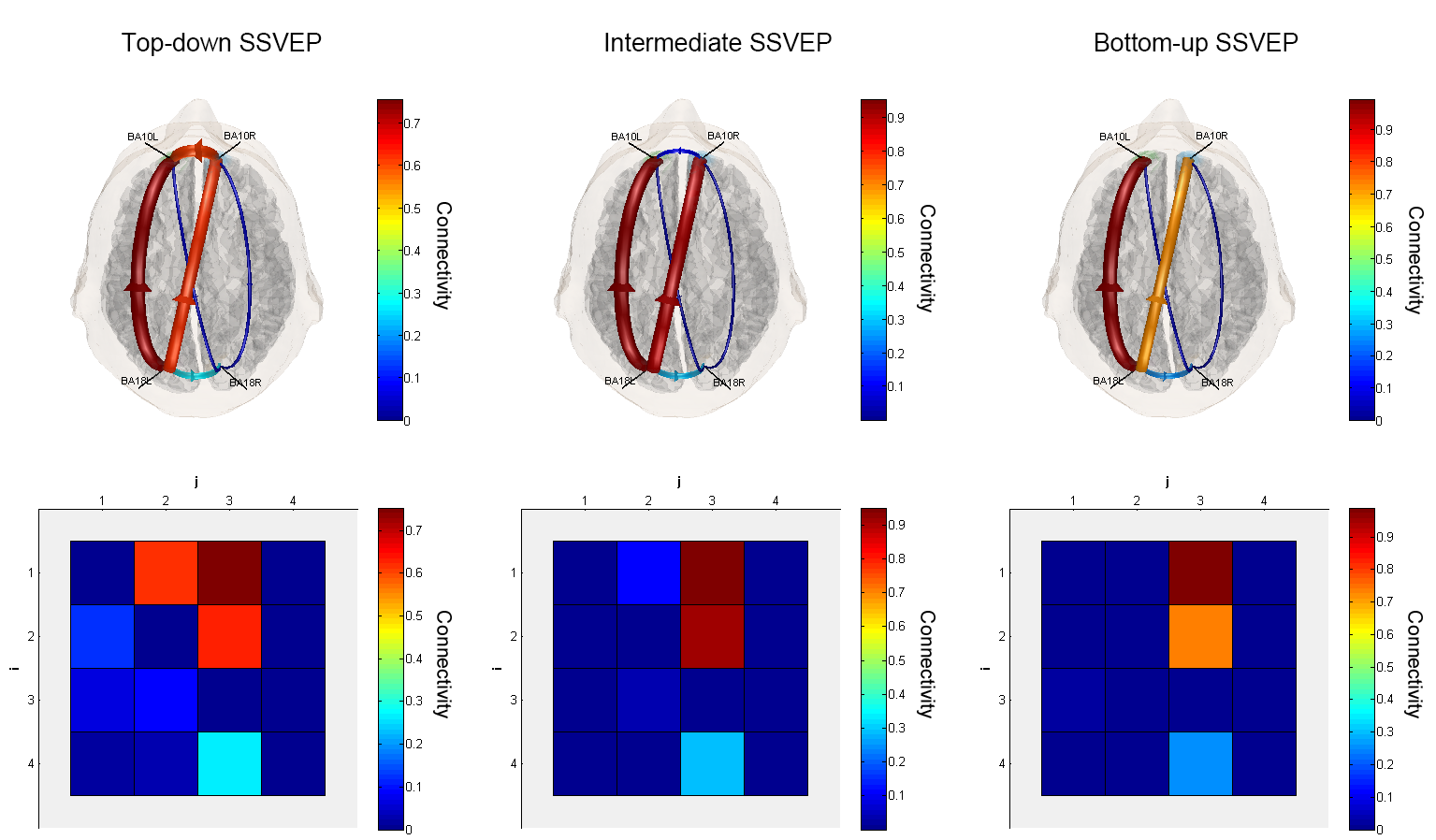


| Supplementary Figure S11. Time-reversed Granger causal connectivity |  |  |
| --- | --- | --- |

1. **Video clips of the three SSVEP conditions and** **online implementation**

Four video clips were included as supplementary information. Video clips 1–3 are examples of the top-down, intermediate, and bottom-up SSVEP stimuli, respectively. Regarding video clips 1 (the top-down SSVEP stimulus) and 3 (the bottom-up SSVEP stimulus), any letter among the six Korean letters could be randomly cued, thus it is not informative to provide a sample cue for these video clips. However, for video clip 2 (the intermediate SSVEP stimulus), the letter ‘ㄱ’ was cued as an example.

The first online test (video clip 4) has confirmed its feasibility as a real-time application, but will require further detailed studies of robustness and user fatigue for the development of an online system with maximal performance. Using a 4-fold cross-validation method^2^, new untrained EEG trials in the prediction phase were classified based on extracted (hyper-) parameters from labelled training EEG trials in the calibration phase. In video clip 4, the right-side monitor presented the predicted letter by the classifier using online EEG signals. When the prediction was matched with the cued letter by an analogue instruction sound, feedback was presented below the predicted letter (the word “Success!” in red). If not, the word “Failure” in blue was presented.

**References**

1 Blankertz, B., Lemm, S., Treder, M., Haufe, S. & Müller, K.-R. Single-trial analysis and classification of ERP components—a tutorial. *NeuroImage* **56**, 814-825 (2011).

2 Lemm, S., Blankertz, B., Dickhaus, T. & Müller, K.-R. Introduction to machine learning for brain imaging. *NeuroImage* **56**, 387-399 (2011).

3 Lin, Z., Zhang, C., Wu, W. & Gao, X. Frequency recognition based on canonical correlation analysis for SSVEP-based BCIs. *IEEE Trans. Biomed. Eng.* **54**, 1172-1176, doi:10.1109/TBME.2006.886577 (2007).

4 Bin, G., Gao, X., Yan, Z., Hong, B. & Gao, S. An online multi-channel SSVEP-based brain-computer interface using a canonical correlation analysis method. *J. Neural Eng.* **6**, 046002, doi:10.1088/1741-2560/6/4/046002 (2009).

5 Krepki, R., Blankertz, B., Curio, G. & Muller, K. R. The Berlin Brain-Computer Interface (BBCI) - towards a new communication channel for online control in gaming applications. *Multimed. Tools Appl.* **33**, 73-90, doi:10.1007/s11042-006-0094-3 (2007).

6 Winkler, I., Panknin, D., Bartz, D., Müller, K.-R. & Haufe, S. Validity of time reversal for testing Granger causality. *IEEE Trans. Signal Process.* **64**, 2746-2760 (2016).

7 Haufe, S., Nikulin, V. V., Muller, K. R. & Nolte, G. A critical assessment of connectivity measures for EEG data: A simulation study. *NeuroImage* **64**, 120-133, doi:10.1016/j.neuroimage.2012.09.036 (2013).
